# Supplementary material for: Depressed mood as a transdiagnostic target relevant to anxiety and/or psychosis: a scoping review protocol
Source: BMJ Open. 2024 May 30;14(5):e077695. doi: 10.1136/bmjopen-2023-077695 (PMC11141174; doi:10.1136/bmjopen-2023-077695)
Supplement: Supplementary data [file bmjopen-2023-077695supp002.pdf]

S1 Table: Additional search strategies

**Population**	Adults
**Exposure/conditions**	*Depressed mood NOT depression AND (psychosis OR anxiety)
**Interventions**	Any, e.g. pharmacological, psychological, physical, etc<br>* to run a generic approach at first and then run subsequent searches specifying the intervention type
**Comparison**	Any, i.e. control, treatment as usual
**Time**	2004 up to search penultimate date
**Setting**	Unrestricted
**Study designs**	Quantitative, i.e. cross-sectional, case-control, cohort, experimental designs (e.g. clinical trials, quasi-experimental designs)
**Language**	English only
