# Supplementary material for: Depressed mood as a transdiagnostic target relevant to anxiety and/or psychosis: a scoping review protocol
Source: BMJ Open. 2024 May 30;14(5):e077695. doi: 10.1136/bmjopen-2023-077695 (PMC11141174; doi:10.1136/bmjopen-2023-077695)
Supplement: Author's manuscript [file bmjopen-2023-077695.draft_revisions.pdf]

BMJ Open is committed to open peer review. As part of this commitment we make the peer review history of every article we publish publicly available.

When an article is published we post the peer reviewers' comments and the authors' responses online. We also post the versions of the paper that were used during peer review. These are the versions that the peer review comments apply to.

The versions of the paper that follow are the versions that were submitted during the peer review process. They are not the versions of record or the final published versions. They should not be cited or distributed as the published version of this manuscript.

BMJ Open is an open access journal and the full, final, typeset and author-corrected version of record of the manuscript is available on our site with no access controls, subscription charges or pay-per-view fees (http://bmjopen.bmj.com).

If you have any questions on BMJ Open's open peer review process please email info.bmjopen@bmj.com

BMJ Open

Depressed mood as a transdiagnostic target relevant to anxiety and/or psychosis: a scoping review protocol

Journal:	*BMJ Open*
Manuscript ID	bmjopen-2023-077695
Article Type:	Protocol
Date Submitted by the Author:	12-Jul-2023
Complete List of Authors:	Dambi, Jermaine; Faculty of Medicine and Health Sciences, Department of Primary Health Care Sciences<br>Mavindidze, Edwin; Faculty of Medicine and Health Sciences, Department of Primary Health Care Sciences<br>Nyamayaro, Primrose; University of Zimbabwe, Department of Primary Health Care Sciences, Unit of Mental Health<br>Beji-Chauke, Rhulani; Friendship Bench<br>Tunduwani, Tariro ; Faculty of Medicine and Health Sciences, Department of Primary Health Care Sciences<br>Shava, Beatrice K.; Faculty of Medicine and Health Sciences, Department of Primary Health Care Sciences<br>Mavhu, Webster; Centre for Sexual Health and HIV/AIDS Research; Liverpool School of Tropical Medicine<br>Abas, Melanie; King's College London, Health Service and Population Research Department<br>Chibanda, Dixon; Friendship Bench ; London School of Hygiene & Tropical Medicine<br>NHUNZVI, CLEMENT; Faculty of Medicine and Health Sciences, Department of Primary Health Care Sciences; Bond University Faculty of Health Sciences and Medicine
Keywords:	Anxiety disorders < PSYCHIATRY, Depression & mood disorders < PSYCHIATRY, Schizophrenia & psychotic disorders < PSYCHIATRY, Psychometrics

SCHOLARONE™
Manuscripts

Title: Depressed mood as a transdiagnostic target relevant to anxiety and/or psychosis: a scoping review protocol

Authors: Jermaine M. Dambi [1,2, § ‡], Edwin Mawindidze [1 ‡], Primrose Nyamayaro [3], Rhulani Beji-Chauke [2], Tariro D. Tunduwani [1], Beatrice K. Shava [1,2], Webster Mavhu [4,5], Melanie Abas [6], Dixon Chibanda [2,3,7], Clement Nhunzvi [1,8]

Affiliations:

[1] University of Zimbabwe: Rehabilitation Sciences Unit, Department of Primary Health Care Sciences, Faculty of Medicine, and Health Sciences. PO Box AV 178 Avondale, Harare, Zimbabwe

[2] Friendship Bench, 4 Weale Road, Milton Park, Harare, Zimbabwe

[3] Mental Health Unit, Depart of Primary Health Care Sciences Faculty of Medicine, and Health Sciences. PO Box AV 178 Avondale, Harare, Zimbabwe

[4] Centre for Sexual Health and HIV/AIDS Research (CeSHHAR), 4 Bath Harare, Zimbabwe

[5] Liverpool School of Tropical Medicine, Pembroke Pl, Liverpool L3 5QA, United Kingdom

[6] Health Service and Population Research Department, Institute of Psychiatry, Psychology and Neuroscience, King's College London, London SE5 8AF, United Kingdom

[7] London School of Hygiene & Tropical Medicine, Keppel St, London WC1E 7HT, United Kingdom

[8] Faculty of Health Sciences and Medicine, Bond University, Gold Coast Queensland, 4229, Australia

§ Corresponding author

‡ Joint first co-authors

Email addresses:

Jermaine M. Dambi (JMD): jermainedambi@gmail.com/jermainedambi@friendshipbench.io
Edwin Mavindidze (EM): eddie.mavie@gmail.com
Primrose Nyamayaro (PM): primrose.nyamayaro@gmail.com
Tariro DeeTunduwani (TDT): tarirodeetunduwani@gmail.com
Beatrice Kudzaishe Shava (BKS):beakshava@gmail.com
Rhulani Beji-Chauke (RBC): rhulani.chauke@friendshipbench.io
Webster Mavhu (WM): webster@ceshhar.co.zw
Melanie Abas (MA): melanie.abas@kcl.ac.uk
Dixon Chibanda (DC): dixon.chibanda@friendshipbench.io
Clement Nhunzvi (CN): clemynhu@gmail.com
Word count: 1989 words

For peer review only

Abstract

Introduction: Depressed mood is a psychological state characterised by sadness, irritability, emptiness, or loss of pleasure or interest in activities. Depressed mood is a highly prevalent early sign and symptom across major mental disorders. However, there is limited understanding of the burden and management of comorbid depressed mood across major mental disorders. Therefore, this scoping review aims to summarise knowledge on depressed mood as a transdiagnostic target among persons with anxiety and/or psychosis. The specific aims are to:

1) Describe the epidemiology and risk factors of depressed mood as a transdiagnostic target among persons with anxiety and/or psychosis.
2) Identify commonly used outcome measures for depressed mood and outline initial evidence of psychometric robustness.
3) Identify and summarise the effectiveness of commonly applied depressed mood modification interventions.

Methods: This scoping review will be conducted per Arksey and O'Malley's framework. We will first search articles in peer-reviewed articles and grey literature published from 2004 to 2023 in databases such as PubMed Scopus, Web of Science, Africa- Wide Information, CINAHL, PsychINFO, and Google Scholar. Afterwards, two researchers will screen articles by title and abstract before data extraction commences. Two independent researchers will do data extraction. Last, we will collate, analyse and chart data in consultation with researchers with lived experiences of depressed mood.

Discussion: The proposed review will provide insights into the burden and management of depressed mood in persons with anxiety and psychosis. The information will inform the development of targeted transdiagnostic interventions.

Keywords: anxiety, depressed mood, psychosis, scoping review & transdiagnostics.

Strengths and limitations of this study:

- Use of robust sscoping review framework
- Duplicate artcle screening and data collection
- Involvement of persons with lived experiences of depressed mood
- Language bias possibility

For peer review only

Introduction

Depressed mood is a temporary psychological state characterised by sadness, irritability, emptiness, unhappiness, or loss of pleasure/interest in activities [1,2]. Although depressed mood can sometimes lead to depression or other mental conditions, it differs from clinical depression as it is a fleeting psychological state that can resolve naturally without treatment [3]. Depressed mood is a highly prevalent early sign and symptom across major mental disorders, including mood disorders (e.g. depression and bipolar affective disorders), psychosis, anxiety disorders, substance use disorders and personality disorders [1,2,4]. A recent meta-analysis showed the odds of depressed mood in persons with generalised anxiety disorders to be up to 12 times (95% CI 5.2-26.3) [2]. Also, up to 23% of persons experiencing first-episode psychosis suffer from depression [1].

Diagnosing mental conditions is based on observations of latent constructs (signs and symptoms). The exact aetiology of depressed mood in patients with comorbid mental conditions such as anxiety and psychosis is unknown; several theories have been postulated [4,5]. For example, the network theory of mental disorders posits that co-morbidity is due to concurring symptoms [5]. Co-existing symptoms have a compound effect, i.e., the more significant the co-occurrence, the greater the morbidity [1,5]. For instance, the association between depression and anxiety is bidirectional, i.e., they are mutual bidirectional risk factors [2,4]. Patients with depression are at increased risk of developing anxiety, with the reverse also true. Depressed mood and psychosis are also mutual bidirectional risk factors [1]. In persons with psychosis, comorbid anxiety/depression is often associated with severe symptoms, poor prognosis and increased psychotic experiences [1].

There is a great propensity towards transdiagnostic interventions to mitigate the enormous mental health care gap, including addressing depressed mood [6]. Consequently, Wellcome Trust has coined the term transdiagnostic target to imply any co-occurring symptoms and experiences considered critical by people with lived experiences as "best bets" for intervention with a high potential return-on-investment and downstream improved health-related quality of life (HRQoL) and functioning in people with mental health conditions [7]. Of the multiple mental disorder symptoms, depressed mood is a significant transdiagnostic target with multilevel impacts. Depressed mood leads to cognitive difficulties (e.g. poor attention and concentration), emotional exhaustion, feelings of excessive guilt or low self-esteem, sleep challenges, and suicidal thoughts [4,5]. Depressed mood also accounts for physical challenges accompanying mental illnesses, such as changes in appetite or weight and tiredness, low energy and disrupted sleeping patterns [2,8]. Depressed mood, therefore, impairs the achievement of functional and personal goals of those affected, negatively affecting their HRQoL [4]. Lastly, comorbid depressed mood predicts severe morbidity, substance misuse, and treatment resistance [2,4,5].

Given the multilevel effects of depressed mood, especially if allowed to progress, early detection, referral and management are critical. Early screening is only contingent upon the availability of validated screening tools. Depressed mood is measurable, and several screening tools are available, but their evidence of diagnostic accuracy is fragmented. For example, the patient health questionnaire (PHQ-9) is one of the most commonly used tools, with evidence of diagnostic accuracy and psychometric performance across systematic reviews [9,10]. The PHQ-9, like other screening tools, has been used globally across different contexts (clinical or research) with comparable transcultural validity and diagnostic performance [9,10]. Screening tools are often used conjunctively with clinical diagnostic interviews to ascertain the clinical diagnosis to guide treatment. However, due to human resources shortages, screening results can inform treatment without a confirmatory clinical diagnostic interview. Fortunately, there are scalable, effective, low-cost interventions for managing depressed mood across socio-economic contexts. For example, Friendship Bench is an evidence-based, task-shifting intervention effective in managing depressed mood [11]. The Friendship Bench has been successfully implemented in a low-income country (Zimbabwe) and is being scaled up globally (www.friendshipbenchzimbabwe.org). Early interventions targeting depressed mood can have far-reaching and sustainable outcomes. As with other mental conditions/psychological states, early treatment should presumably increase treatment engagement, resulting in improved HRQoL, increased functioning and community participation [1,2,4]. Managing depressed mood should give the affected person a fair chance to engage and benefit from other treatments requiring active participation and increased pleasure and interest in activities. However, there is limited understanding of the burden and management of comorbid depressed mood across major mental disorders. Therefore, this review aims to summarise knowledge on depressed mood as a transdiagnostic target among persons with anxiety and/or psychosis. The specific aims are to:

For peer review only - http://bmjopen.bmj.com/site/about/guidelines.xhtml

1) Describe the epidemiology and risk factors of depressed mood as a transdiagnostic target among persons with anxiety and/or psychosis.
2) Identify commonly used outcome measures for depressed mood and outline initial evidence of psychometric robustness.
3) Identify and summarise the effectiveness of commonly applied depressed mood modification interventions.

Methods

Overview

To review and summarise the current knowledge base for depressed mood among those with anxiety and/or psychosis, we will conduct a scoping review (SR). The SR will be conducted per Arksey and O'Malley's framework [12]. A scoping review aims to identify the available evidence on a particular concept or subject [13]. Below, we describe the SR methodology guided by the six stages of Arksey and O'Malley's framework.

Stage 1: Identifying the research question(s)

To address the study's aim, the scoping review seeks to answer these questions:

1) What is the epidemiology and risk factors of depressed mood in persons with anxiety and/or psychosis?
2) What are commonly used outcome measures used for depressed mood?
3) What is the collective evidence of psychometric robustness of commonly used depressed mood outcome measures?
4) What approaches and interventions are used to modify depressed mood?
5) How effective are commonly applied depressed mood modification interventions?

The research team will engage in an extensive and iterative process of consultation and interactions with various stakeholders, including persons with lived experience of anxiety and/or psychosis, to refine the review questions further. Once the research questions are finalised, the team will refine keywords that will guide the search strategy.

Stage 2: Identifying relevant studies.

The goal is to search broadly enough to capture all the available evidence that answers the study question by producing meaningful results [14]. Through iterative cycles of preliminary searches and refinement, the team developed provisional inclusion criteria as outlined below.

Study inclusion criteria

- Peer-reviewed articles, grey literature, original research and conceptual papers will be included.
- Only literature published in English. We do not have the financial resources to translate and analyse studies published in other languages.
- Literature published from 2004 to 2023. The year 2004 was selected as the start date as quick pilot searches show it to be the year the first article on transdiagnostic targets was published.
- Literature that reports on the identification and management of depressed mood across various conditions. For example, psychosis, anxiety, and other mental conditions.

Search strategy

Key search terms will include "depressed mood and its variants", "transdiagnostic", and "psychosis and its variants", and "anxiety and its alternative terms". Table 1 outlines an example search strategy for the CINHAL and PubMed databases. We will apply a combination of Boolean logic operators to glean the literature.

<Insert Table 1 here>

For peer review only - http://bmjopen.bmj.com/site/about/guidelines.xhtml

Table 1: CINHAL & PubMed search strategy

Key search terms	Alternative terms	PubMed
Depressed mood	Depressive symptoms OR Depressive affect OR Depress*	Depression [MeSH]
Transdiagnostic		Transdiagnostic
Psychosis	Psychotic disorder OR Psychoses OR Psycho* OR Schizophreni* (For schizophreniform, Schizophrenia)<br>Schizoaffective OR Brief reactive psychosis OR Substance-induced psychoses	Psychotic Disorders [MeSH] OR Psychoses OR Substance-induced [MeSH]
Anxiety	Social anxiety OR Nervousness OR Anxiousness OR<br>Anxiety Disorder OR Anxi* OR Neurotic OR Neuroses	Anxiety [MeSH] OR Anxiety Disorders [MeSH]

For peer review only

Databases

Utilising keywords, two researchers (JD&EM) will conduct broad searches in these electronic databases: PubMed, Academic Search Premier, Africa-Wide Information, the Cumulative Index to Nursing and Allied Health Literature (CINAHL), Humanities International Complete, Psych Info, Sabinet, SocINDEX, Scopus, Web of Science and Open Grey. These databases have been chosen based on expert advice and their relevance to the research questions. Guided by an expert librarian, we conducted preliminary searches on PubMed, refined key search terms and identified MeSH terms. We further customised the search strategy for the other databases.

Stage 3: Study Selection.

Data management

Once the literature search is complete, the citations will be uploaded to Rayyan [15] to prepare for the study selection process.

Screening

Two review teams (Team 1 JD and PM; Team 2 RC and EM) will screen the retrieved articles in three steps. First, both teams will independently review citations to identify articles for inclusion. The articles will also be assessed to determine whether they meet the selection criteria at both the title and abstract levels. At this preliminary stage, the goal is to identify articles focusing on depressed mood as a transdiagnostic target. If there is uncertainty, the article will be further reviewed in the full article screening step. The included articles will be captured on an Excel spreadsheet, and the lists from the two teams will be compared. In cases of reviewers' disagreement(s), a consensus meeting will be convened, where the article will be further reviewed, and a decision will be agreed upon. Full texts of included studies will be retrieved to prepare for data extraction.

Stage 4: Charting the data.

Two researchers not involved in the screening process (CN and WM) will independently review and read the full articles and rate them against the inclusion criteria. Where reviewers do not agree, a consensus meeting will be held to agree on a decision. Throughout all three steps, Cohen's kappa will be calculated to assess the level of agreement between reviewers. If the level of agreement is weak ($\kappa < 0.60$), the process will be repeated until there is a strong agreement. This stage aims to contextualise the study findings by briefly describing each article, including the methodology used to reach conclusions [12]. Bibliometric information will include the authors, year of publication, country of study, the study design, study population and essential results. Data will be captured in Microsoft Excel.

Stage 5: Collating, summarising and reporting the results.

The extracted data will be collated and presented in a narrative synthesis with a tabular supplement [16]. Through numerical analysis, we seek to produce charts and tables highlighting the distribution of

For peer review only - http://bmjopen.bmj.com/site/about/guidelines.xhtml

the findings concerning geographical distribution, distribution across diagnoses, distribution across the years and the nature of the reported studies. In addition, the results will be presented thematically in line with the study objectives. We will qualitatively summarise the epidemiology of transdiagnostic depressed mood, its outcome measures, interventions and natural progression in anxiety and psychosis.

Stage 6: Consultation Exercise.

Before compiling a final report, we will present our preliminary findings to various stakeholders, including individuals with lived experience, clinicians, and researchers. Before presenting the preliminary findings, the research team will orient the stakeholders on the scoping review objectives. This will help gain more insight into the meaning of the results and guide the research team towards resources and evidence that might have been missed through the search strategy in preparation for the final report. Additionally, this stage will serve as a precursor to disseminating study findings. We will share the dissemination strategy and use the feedback from the stakeholders to refine the strategy.

Patient and Public Involvement Statement

The experiential meaning and impact of depressed mood as a transdiagnostic target can be further explored through the narratives of those with lived experience. We will conduct extensive and iterative processes of consultation and interaction with various stakeholders, including persons with lived experience of anxiety and/or psychosis, to refine the review questions. We plan to triangulate insights from our SR with contextual data from key informants, including persons with lived experience, clinicians, and researchers. We will use purposive sampling to reach information-rich informers and engage in two rounds of focus group discussions and in-depth interviews.

Ethics and dissemination

The review will glean evidence regarding the potential and possible socio-economic and health benefits when depressed mood is prioritised and targeted. We will contribute insight into the contextual realities of depressed mood as a transdiagnostic target for persons with mental disorders in low-to-middle-income countries. The review will also identify evidence gaps and recommendations for future research.

Declarations

Ethics approval and consent to participate.

Not applicable.

Consent for publication

Not applicable.

Availability of data and materials

Not applicable to a review protocol. However, all relevant data/materials, including data collection tools, will be submitted as supplementary files during the publication of the full review.

Competing interests

All the authors declare no competing interests.

Authors' contributions

JMD and EM were primarily responsible for writing the protocol protocol first draft. JMD, EM, PN, RBC, TT, BKS, MA, DC, WM and CN were involved in conceptualising the study and editing all protocol manuscript versions. JMD will search the literature and data management. EM, PN, RBC, TDT, BKS, MA, DC, WM and CN will be responsible for article screening and quality assurance, data extraction and qualitative synthesis. MA, WM, CN and DC will provide overall supervision, mentoring and guidance.

For peer review only

Authors' ORCID information

- Jermaine M. Dambi (JMD): https://orcid.org/0000-0002-2446-7903
- Edwin Mawindidze (EM): https://orcid.org/0000-0001-9849-8932
- Primrose Nyamayaro (PN): https://orcid.org/0000-0002-6856-1002
- Tariro DeeTunduwani (TDT): https://orcid.org/0009-0004-8073-7068
- Beatrice Kudzaishe Shava (BKS): https://orcid.org/0000-0002-9130-7010
- Rhulani Beji-Chauke (RBC): https://orcid.org/0000-0002-3157-5507
- Webster Mavhu (WM): https://orcid.org/0000-0003-1881-4398
- Melanie Abas (MA): https://orcid.org/0000-0003-1716-5584
- Dixon Chibanda (DC): https://orcid.org/0000-0003-2505-8607
- Clement Nhunzvi (CN): https://orcid.org/0000-0001-5804-9817

For peer review only

Funding
Wellcome Trust funded this research through the 2023 Wellcome Mental Health Transdiagnostic Targets Review Commission. The funder had no role in study design, data collection, data analysis, data interpretation, or writing of the report.

References

1 Wilson RS, Yung AR, Morrison AP. Comorbidity rates of depression and anxiety in first episode psychosis: A systematic review and meta-analysis. *Schizophr Res* 2020;**216**:322–9. doi:10.1016/j.schres.2019.11.035

2 Saha S, Lim CCW, Cannon DL, *et al.* Co-morbidity between mood and anxiety disorders: A systematic review and meta-analysis. *Depress Anxiety* 2021;**38**:286–306. doi:https://doi.org/10.1002/da.23113

3 Lemstra M, Neudorf C, D'Arcy C, *et al.* A systematic review of depressed mood and anxiety by SES in youth aged 10-15 years. *Can J Public Heal* 2008;**99**:125–9. doi:10.1007/bf03405459

4 Stålner O, Nordin S, Madison G. Six-year prognosis of anxiety and depression caseness and their comorbidity in a prospective population-based adult sample. *BMC Public Health* 2022;**22**:1–9. doi:10.1186/s12889-022-13966-4

5 Kaiser T, Herzog P, Voderholzer U, *et al.* Unraveling the comorbidity of depression and anxiety in a large inpatient sample : Network analysis to examine bridge symptoms. *Depress Anxiety* 2021;**38**:307–17. doi:10.1002/da.23136

6 Zarate-Guerrero S, Duran JM, Naismith I. How a transdiagnostic approach can improve the treatment of emotional disorders: Insights from clinical psychology and neuroimaging. *Clin Psychol Psychother* 2022;**29**:895–905. doi:10.1002/cpp.2704

7 Trust W. Request for Proposals (RFP) for suppliers to review and summarise the current knowledge base for one or more transdiagnostic targets relevant to anxiety , depression and / or psychosis Up to 10 reviews will be commissioned. London: 2022. https://cms.wellcome.org/sites/default/files/2022-11/Wellcome-RFP-Transdiagnostics.pdf

8 Low mood and depression | NHS inform.

9 El-Den S, Chen TF, Gan YL, *et al.* The psychometric properties of depression screening tools in primary healthcare settings: A systematic review. *J Affect Disord* 2018;**225**:503–22. doi:10.1016/j.jad.2017.08.060

10 Levis B, Benedetti A, Thombs BD. Accuracy of Patient Health Questionnaire-9 (PHQ-9) for

For peer review only - http://bmjopen.bmj.com/site/about/guidelines.xhtml

screening to detect major depression: Individual participant data meta-analysis. *BMJ* 2019;**365**. doi:10.1136/bmj.l1476

11 Chibanda D, Weiss HA, Verhey R, *et al.* Effect of a primary care-based psychological intervention on symptoms of common mental disorders in Zimbabwe: A randomized clinical trial. *JAMA - J Am Med Assoc* 2016;**316**:2618–26. doi:10.1001/jama.2016.19102

12 Arksey H, O'Malley L. Scoping studies: towards a methodological framework. *Int J Soc Res Methodol* 2005;**8**:19–32. doi:10.1080/1364557032000119616

13 Peters MDJ, Godfrey C, McInerney P, *et al.* Best practice guidance and reporting items for the development of scoping review protocols. *JBI Evid Synth* 2022;**20**:953–68. doi:10.11124/JBIES-21-00242

14 Samnani SS, Vaska M, Ahmed S, *et al.* Review typology: The basic types of reviews for synthesizing evidence for the purpose of knowledge translation. *J Coll Physicians Surg Pakistan* 2017;**27**:635–41. doi:2724

15 Ouzzani M, Hammady H, Fedorowicz Z, *et al.* Rayyan-a web and mobile app for systematic reviews. *Syst Rev* 2016;**5**:1–10. doi:10.1186/s13643-016-0384-4

16 Mallidou A. Mapping the landscape of knowledge synthesis. *Nurs Manage* 2014;**21**:30–9. doi:10.7748/nm.21.5.30.e1242

For peer review only

Preferred Reporting Items for Systematic reviews and Meta-Analyses extension for Scoping Reviews (PRISMA-ScR) Checklist

SECTION	ITEM	PRISMA-ScR CHECKLIST ITEM	REPORTED ON PAGE #
**TITLE**			
Title	1	Identify the report as a scoping review.	1
**ABSTRACT**			
Structured summary	2	Provide a structured summary that includes (as applicable): background, objectives, eligibility criteria, sources of evidence, charting methods, results, and conclusions that relate to the review questions and objectives.	2
**INTRODUCTION**			
Rationale	3	Describe the rationale for the review in the context of what is already known. Explain why the review questions/objectives lend themselves to a scoping review approach.	3-4
Objectives	4	Provide an explicit statement of the questions and objectives being addressed with reference to their key elements (e.g., population or participants, concepts, and context) or other relevant key elements used to conceptualize the review questions and/or objectives.	3-4
**METHODS**			
Protocol and registration	5	Indicate whether a review protocol exists; state if and where it can be accessed (e.g., a Web address); and if available, provide registration information, including the registration number.	N/A
Eligibility criteria	6	Specify characteristics of the sources of evidence used as eligibility criteria (e.g., years considered, language, and publication status), and provide a rationale.	4
Information sources*	7	Describe all information sources in the search (e.g., databases with dates of coverage and contact with authors to identify additional sources), as well as the date the most recent search was executed.	5
Search	8	Present the full electronic search strategy for at least 1 database, including any limits used, such that it could be repeated.	4-5
Selection of sources of evidence†	9	State the process for selecting sources of evidence (i.e., screening and eligibility) included in the scoping review.	5
Data charting process‡	10	Describe the methods of charting data from the included sources of evidence (e.g., calibrated forms or forms that have been tested by the team before their use, and whether data charting was done independently or in duplicate) and any processes for obtaining and confirming data from investigators.	5-6
Data items	11	List and define all variables for which data were sought and any assumptions and simplifications made.	5-6
Critical appraisal of individual sources of evidence§	12	If done, provide a rationale for conducting a critical appraisal of included sources of evidence; describe the methods used and how this information was used in any data synthesis (if appropriate).	N/A
Synthesis of results	13	Describe the methods of handling and summarizing the data that were charted.	6

For peer review only

SECTION	ITEM	PRISMA-ScR CHECKLIST ITEM	REPORTED ON PAGE #
**RESULTS**			
Selection of sources of evidence	14	Give numbers of sources of evidence screened, assessed for eligibility, and included in the review, with reasons for exclusions at each stage, ideally using a flow diagram.	n/a
Characteristics of sources of evidence	15	For each source of evidence, present characteristics for which data were charted and provide the citations.	N/A
Critical appraisal within sources of evidence	16	If done, present data on critical appraisal of included sources of evidence (see item 12).	N/A
Results of individual sources of evidence	17	For each included source of evidence, present the relevant data that were charted that relate to the review questions and objectives.	N/A
Synthesis of results	18	Summarize and/or present the charting results as they relate to the review questions and objectives.	N/A
**DISCUSSION**			
Summary of evidence	19	Summarize the main results (including an overview of concepts, themes, and types of evidence available), link to the review questions and objectives, and consider the relevance to key groups.	7
Limitations	20	Discuss the limitations of the scoping review process.	N/A
Conclusions	21	Provide a general interpretation of the results with respect to the review questions and objectives, as well as potential implications and/or next steps.	N/A
**FUNDING**			
Funding	22	Describe sources of funding for the included sources of evidence, as well as sources of funding for the scoping review. Describe the role of the funders of the scoping review.	8

JBI = Joanna Briggs Institute; PRISMA-ScR = Preferred Reporting Items for Systematic reviews and Meta-Analyses extension for Scoping Reviews.
* Where *sources of evidence* (see second footnote) are compiled from, such as bibliographic databases, social media platforms, and Web sites.
† A more inclusive/heterogeneous term used to account for the different types of evidence or data sources (e.g., quantitative and/or qualitative research, expert opinion, and policy documents) that may be eligible in a scoping review as opposed to only studies. This is not to be confused with *information sources* (see first footnote).
‡ The frameworks by Arksey and O'Malley (6) and Levac and colleagues (7) and the JBI guidance (4, 5) refer to the process of data extraction in a scoping review as data charting.
§ The process of systematically examining research evidence to assess its validity, results, and relevance before using it to inform a decision. This term is used for items 12 and 19 instead of "risk of bias" (which is more applicable to systematic reviews of interventions) to include and acknowledge the various sources of evidence that may be used in a scoping review (e.g., quantitative and/or qualitative research, expert opinion, and policy document).

From: Tricco AC, Lillie E, Zarin W, O'Brien KK, Colquhoun H, Levac D, et al. PRISMA Extension for Scoping Reviews (PRISMAScR): Checklist and Explanation. Ann Intern Med. 2018;169:467–473. doi: 10.7326/M18-0850.

For peer review only

BMJ Open

Depressed mood as a transdiagnostic target relevant to anxiety and/or psychosis: a scoping review protocol

Journal:	*BMJ Open*
Manuscript ID	bmjopen-2023-077695.R1
Article Type:	Protocol
Date Submitted by the Author:	16-Apr-2024

Complete List of Authors:	Dambi, Jermaine; University of Zimbabwe Faculty of Medicine and Health Sciences, Department of Rehabilitation Sciences ; Friendship Bench<br>Mavindidze, Edwin; University of Zimbabwe Faculty of Medicine and Health Sciences, Department of Rehabilitation Sciences<br>Nyamayaro, Primrose; University of Zimbabwe, Department of Primary Health Care Sciences, Unit of Mental Health<br>Beji-Chauke, Rhulani; Friendship Bench<br>Tunduwani, Tariro ; University of Zimbabwe College of Health Sciences, Department of Rehabilitation Sciences<br>Shava, Beatrice K.; University of Zimbabwe Faculty of Medicine and Health Sciences, Department of Rehabilitation Sciences; Friendship Bench<br>Mavhu, Webster; Centre for Sexual Health and HIV/AIDS Research Zimbabwe; Liverpool School of Tropical Medicine<br>Abas, Melanie; King's College London, Health Service and Population Research Department<br>Chibanda, Dixon; Friendship Bench; London School of Hygiene & Tropical Medicine<br>NHUNZVI, CLEMENT; University of Zimbabwe Faculty of Medicine and Health Sciences, Department of Rehabilitation Sciences; Bond University Faculty of Health Sciences and Medicine

**Primary Subject Heading**:	Mental health
Secondary Subject Heading:	Epidemiology
Keywords:	Anxiety disorders < PSYCHIATRY, Depression & mood disorders < PSYCHIATRY, Schizophrenia & psychotic disorders < PSYCHIATRY, Psychometrics

SCHOLARONE™ Manuscripts

Depressed mood as a transdiagnostic target relevant to anxiety and/or psychosis: a scoping review protocol

Jermaine M. Dambi [1,2, § ‡], Edwin Mavindidze [1 ‡], Primrose Nyamayaro [3], Rhulani Beji-Chauke [2], Tariro D. Tunduwani [1], Beatrice K. Shava [1,2], Webster Mavhu [4,5], Melanie Abas [6], Dixon Chibanda [2,3, 7], Clement Nhunzvi [1,8]

Affiliations:

[1] University of Zimbabwe: Department of Rehabilitation Sciences, Faculty of Medicine, and Health Sciences. PO Box AV 178 Avondale, Harare, Zimbabwe.

[2] Friendship Bench, 4 Weale Road, Milton Park, Harare, Zimbabwe.

[3] University of Zimbabwe: Department of Mental Health, Faculty of Medicine, and Health Sciences. PO Box AV 178 Avondale, Harare, Zimbabwe.

[4] Centre for Sexual Health and HIV/AIDS Research (CeSHHAR), 4 Bath Harare, Zimbabwe.

[5] Liverpool School of Tropical Medicine, Pembroke Pl, Liverpool L3 5QA, United Kingdom.

[6] Health Service and Population Research Department, Institute of Psychiatry, Psychology and Neuroscience, King's College London, London SE5 8AF, United Kingdom.

[7] London School of Hygiene & Tropical Medicine, Keppel St, London WC1E 7HT, United Kingdom.

[8] Faculty of Health Sciences and Medicine, Bond University, Gold Coast Queensland, 4229, Australia

§Corresponding author

‡Joint first co-authors

Email addresses:

Jermaine M. Dambi (JMD): jermainedambi@gmail.com/jermainedambi@friendshipbench.io
Edwin Mavindidze (EM): eddie.mavie@gmail.com
Primrose Nyamayaro (PM): primrose.nyamayaro@gmail.com
Tariro Dee Tunduwani (TDT): tarirodeetunduwani@gmail.com
Beatrice Kudzaishe Shava (BKS): beakshava@gmail.com
Rhulani Beji-Chauke (RBC): rhulani.chauke@friendshipbench.io
Webster Mavhu (WM): webster@ceshhar.co.zw
Melanie Abas (MA): melanie.abas@kcl.ac.uk
Dixon Chibanda (DC): dixon.chibanda@friendshipbench.io
Clement Nhunzvi (CN): clemynhu@gmail.com

Word count: 1989 words

For peer review only

Abstract

Introduction: Depressed mood is a psychological state characterised by sadness or loss of interest in activities. Depressed mood is a highly prevalent symptom across major mental disorders. However, there is limited understanding of the burden and management of comorbid depressed mood across major mental disorders. Therefore, this scoping review aims to summarise knowledge on depressed mood among persons with anxiety and/or psychosis. The specific aims are to describe the epidemiology and risk factors of depressed mood as a transdiagnostic target among persons with anxiety and/or psychosis, to identify commonly used outcome measures for depressed mood and outline initial evidence of psychometric robustness and to identify and summarise the effectiveness of commonly applied depressed mood modification interventions. Our hope is that the proposed review will provide insights into the burden of depressed mood in persons with anxiety and psychosis and help to identify evidence gaps and recommendations for future research.

Methods and analysis: This scoping review will be conducted per Arksey and O'Malley's framework. We will first search for peer-reviewed articles and grey literature published from 2004 to 2023 in PubMed, Scopus, Web of Science, Africa-Wide Information, CINAHL, PsycINFO, Academic Search Premier, Humanities International Complete, Sabinet, SocINDEX, Open Grey and Google Scholar. We will include articles reporting depressed mood (subthreshold depression) among persons with anxiety and/or psychosis. Studies recruiting participants meeting depression diagnostic criteria and those published in non-English languages will be excluded. Two independent researchers will extract the data. We will analyse and chart data collaboratively with researchers with lived experiences of depressed mood.

Ethics and dissemination: This study does not require ethical approval as it is a literature review. The results will be submitted for publication in a peer-reviewed journal.

Keywords: anxiety, depressed mood, psychosis, scoping review, transdiagnostics.

Strengths and limitations of this study

- Use of robust scoping review framework.
- Duplicate article screening and data collection.
- Involvement of persons with lived experiences of depressed mood.
- Possibility of language bias.

For peer review only

INTRODUCTION

Depressed mood is a temporary psychological state characterised by sadness, irritability, emptiness, unhappiness, or loss of pleasure/interest in activities [1,2]. Although depressed mood can sometimes lead to depression or other mental disorders, it differs from clinical depression as it is a fleeting psychological state that can resolve naturally without treatment [3]. Depressed mood is a highly prevalent early sign and symptom across major mental disorders, including mood disorders (e.g. depression and bipolar affective disorders), psychosis, anxiety disorders, substance use disorders and personality disorders [1,2,4]. A recent meta-analysis showed the odds of depressed mood in persons with generalised anxiety disorders to be up to 12 times (95% CI 5.2-26.3) as compared to persons without generalised anxiety disorders [2]. Also, up to 23% of persons experiencing first-episode psychosis suffer from depression [1].

Diagnosing mental disorders is based on observations of latent constructs (signs and symptoms). The exact aetiology of depressed mood in patients with comorbid mental disorders such as anxiety and psychosis is unknown; several theories have been postulated [4,5]. For example, the network theory of mental disorders posits that co-morbidity is due to concurring symptoms [5]. Co-existing symptoms have a compound effect, i.e., the more significant the co-occurrence, the greater the morbidity [1,5]. For instance, the association between depression and anxiety is bidirectional, i.e., they are mutual bidirectional risk factors [2,4]. Patients with depression are at increased risk of developing anxiety, with the reverse also true. Depressed mood and psychosis are also mutual bidirectional risk factors [1]. In persons with psychosis, comorbid anxiety/depression is often associated with severe symptoms, poor prognosis and increased psychotic experiences [1]. The high prevalence of co-existing symptoms across mental disorders has inherently led to an inclination towards the utilisation of transdiagnostic interventions.

The transdiagnostic treatment approach is premised on interventions designed to treat co-occurring symptoms simultaneously [6]. The transdiagnostic treatment approach can mitigate the enormous mental healthcare gap by addressing depressed mood and several mental disorders. Globally, there is a substantial mental healthcare gap, i.e. there is a huge mismatch in the high number of people in need of mental healthcare against the few available treatment/care resources [7]. The care gap spans all mental disorders, including depressed mood, which is highly prevalent [8,9]. Consequently, transdiagnostic interventions can potentially mitigate the care gap as multimodal interventions, such as the WHO Mental Health Gap Action Programme, have been successfully implemented to address a wide array of mental disorders [7]. Also, Wellcome Trust has coined the term transdiagnostic target to imply any co-occurring symptoms and experiences considered critical by people with lived experiences as "best bets" for intervention with a high potential return-on-investment and downstream improved health-related quality of life (HRQoL) and functioning in people with mental health disorders [10]. Of the multiple mental disorder symptoms, depressed mood is a significant transdiagnostic target with multilevel impacts. Depressed mood, a precursor to depression and other mental conditions, often leads to cognitive difficulties (e.g. poor attention and concentration), emotional exhaustion, feelings of excessive guilt or low self-esteem, sleep challenges, and suicidal thoughts; classical depression signs and symptoms [4,5]. Depressed mood also accounts for physical challenges accompanying mental illnesses, such as changes in appetite or weight, tiredness, low energy, and disrupted sleeping patterns [2,11]. Depressed mood, therefore, impairs the achievement of functional and personal goals of those affected, negatively affecting their HRQoL [4]. Lastly, comorbid depressed mood predicts severe morbidity, substance misuse, and treatment resistance [2,4,5].

Given the multilevel effects of depressed mood, especially if allowed to progress, early detection, referral and management are critical. Early screening is contingent upon the availability of validated screening tools. Depressed mood is measurable, and several screening tools are available, but their evidence of diagnostic accuracy is fragmented. For example, the patient health questionnaire (PHQ-9) is one of the most commonly used tools, with evidence of diagnostic accuracy and psychometric performance across systematic reviews [12,13]. The PHQ-9, like other screening tools, has been used globally across different contexts (clinical or research) with comparable transcultural validity and diagnostic performance [12,13]. Screening tools are often used conjunctively with clinical diagnostic interviews to ascertain the clinical diagnosis and guide treatment. However, due to human resources shortages, screening results can inform treatment without a confirmatory clinical diagnostic interview.

For peer review only - http://bmjopen.bmj.com/site/about/guidelines.xhtml

Fortunately, there are scalable, effective, low-cost interventions for managing depressed mood across socio-economic contexts. For example, Friendship Bench is an evidence-based, task-shifting intervention effective in managing depressed mood [14]. The Friendship Bench has been successfully implemented in a low-income country (Zimbabwe) and is being scaled up globally.
Furthermore, physical modalities such as exercise have been proven to be effective in mitigating the effects of depressed mood across all age groups [15]. For instance, a recent meta-analysis showed that exercise had moderate effects on depressed mood [16]. Therefore, interventions that include exercise can be a low-hanging fruit that can potentially reduce the progression of depressed mood. Early interventions targeting depressed mood can have far-reaching and sustainable outcomes. As with other mental conditions/psychological states, early treatment should presumably increase treatment engagement, resulting in improved HRQoL, increased functioning and community participation [1,2,4]. Managing depressed mood should give the affected person a fair chance to engage and benefit from other treatments requiring active participation and increased pleasure and interest in activities. However, there is limited understanding of the burden and management of comorbid depressed mood across major mental disorders. Therefore, this review aims to summarise knowledge on depressed mood as a transdiagnostic target among persons with anxiety and/or psychosis. The specific aims are to:

1) Describe the epidemiology and risk factors of depressed mood as a transdiagnostic target among persons with anxiety and/or psychosis.
2) Identify commonly used outcome measures for depressed mood and outline initial evidence of psychometric robustness.
3) Identify and summarise the effectiveness of commonly applied depressed mood modification interventions.

Our hope is that the proposed review will provide insights into the burden of depressed mood in persons with anxiety and psychosis and help to identify evidence gaps and recommendations for future research.

METHODS AND ANALYSIS

Overview

We will conduct a scoping review (SR) to review and summarise the current knowledge base on depressed mood among those with anxiety and/or psychosis. The SR will be conducted per Arksey and O'Malley's framework [17]. A scoping review aims to identify the available evidence on a particular concept or subject [18]. The review is reported per referred Reporting Items for Systematic reviews and Meta-Analyses extension for Scoping Reviews (PRISMA-ScR) [19] - see Additional File 1. Below, we describe the SR methodology guided by the six stages of Arksey and O'Malley's framework.

Stage 1: Identifying the research question(s)

In the context of depressed mood, this scoping aims to seek to answer these questions:

1) What is the epidemiology and risk factors of depressed mood in persons with anxiety and psychosis?
2) What are commonly used outcome measures used for depressed mood in persons with anxiety and psychosis?
3) What is the collective evidence of psychometric robustness of commonly used depressed mood outcome measures in persons with anxiety and psychosis?
4) What approaches and interventions are used to modify depressed mood in persons with anxiety and psychosis?
5) In persons with anxiety and psychosis, how effective are commonly applied depressed mood modification interventions?

The research team engaged in an extensive and iterative process of consultation and interactions with various stakeholders, including persons with lived experience of anxiety and/or psychosis, to refine the review questions further. Once the research questions are finalised, the team will refine keywords that will guide the final search strategy.

For peer review only - http://bmjopen.bmj.com/site/about/guidelines.xhtml

Stage 2: Identifying relevant studies

The goal is to search broadly enough to capture all the available evidence that answers the study question by producing meaningful results [20]. Through iterative cycles of preliminary searches and refinement, the team developed the inclusion criteria outlined below.

Study inclusion and exclusion criteria

For this review, we will include:

- Peer-reviewed articles, grey literature, original research, and conceptual papers will be included. There will be no restrictions on the type of study designs and settings included to attain literature saturation.
- We will include literature published in English. We do not have the financial resources to translate and analyse studies published in other languages.
- Literature published from 2004 to 2023. The year 2004 will be selected as the start date as quick pilot searches show it to be the year the first article on transdiagnostic targets was published.
- Literature that reports on the identification and/or management of comorbid depressed mood in patients with anxiety and or psychosis. We will include anxiety disorders per DSM 5 criteria, including generalised anxiety, panic disorder, social anxiety disorder, and specific phobias. We excluded anxiety disorders due to medications or other medical conditions and obsessive-compulsive disorders. For psychosis, we will include those meeting the diagnostic criteria of psychotic disorder as specified in DSM 5, characterised by delusions, hallucinations, disorganised thinking, and negative symptoms. Psychosis diagnoses include Schizophrenia, delusional disorders, reactive psychosis, substance-induced psychosis, schizoaffective, and schizophreniform. We will similarly exclude psychotic conditions due to neurologic or medical conditions. Also, literature focusing on sub-threshold anxiety and or psychosis will be excluded. We also aim to exclude those studies focusing on clinical depression.

Search strategy

Key search terms included "depressed mood and its variants", "transdiagnostic", and "psychosis and its variants", and "anxiety and its alternative terms". We applied a combination of Boolean logic operators to glean the literature. Table 1 outlines an example search strategy for the CINHAL and PubMed databases. Additional File 2 outlines the search strategy for the rest of the databases.

Table 1. CINHAL & PubMed search strategy

Key search terms	Alternative terms	PubMed
Depressed mood	Depressive symptoms OR Depressive affect OR Depress*	Depression [MeSH]
Transdiagnostic		Transdiagnostic
Psychosis	Psychotic disorder OR Psychoses OR Psycho* OR Schizophreni* (For schizophreniform, Schizophrenia)<br>Schizoaffective OR Brief reactive psychosis OR Substance-induced psychoses	Psychotic Disorders [MeSH] OR Psychoses OR Substance-induced [MeSH]
Anxiety	Social anxiety OR Nervousness OR Anxiousness OR Generalised anxiety disorder OR Panic attack OR phobia<br>Anxiety Disorder OR Anxi* OR Neurotic OR Neuroses	Anxiety [MeSH] OR Anxiety Disorders [MeSH]

Databases

Utilising keywords, two researchers (JD&EM) will conduct broad searches in these electronic databases: PubMed, Academic Search Premier, Africa-Wide Information, the Cumulative Index to Nursing and Allied Health Literature (CINAHL), Humanities International Complete, PsycINFO,

For peer review only - http://bmjopen.bmj.com/site/about/guidelines.xhtml

Sabinet, SocINDEX, Scopus, Web of Science, Open Grey and Google Scholar. These databases were chosen based on expert advice and their relevance to the research questions. Guided by an expert librarian, we will conduct preliminary searches in PubMed, refine key search terms and identify MeSH terms. We will then further customise the search strategy for other databases. Additional File 2 is an outline of the search strategy for the rest of the databases.

Stage 3: Study selection

Data management

Once the literature search is complete, the citations will be uploaded to Rayyan [21] to prepare for the study selection process.

Screening

Two review teams (Team 1 JD and PM; Team 2 RC and EM) will screen the retrieved articles in three steps. First, both teams independently review citations to identify articles for inclusion. The articles will also be assessed to determine whether they meet the selection criteria at both the title and abstract levels. At this preliminary stage, the goal is to identify articles focusing on depressed mood as a transdiagnostic target. If there is uncertainty, the article was further reviewed in the full article screening step. The included articles will be captured on an Excel spreadsheet, and the lists from the two teams compared. In cases of reviewers' disagreement(s), a consensus meeting will be convened, where the article will be further reviewed to make the final decision. Full texts of included studies will be retrieved to prepare for data extraction. Last, we will not perform any quality appraisal of the identified studies. This review aims to landscape and summarise the available evidence on depressed mood, as previous reviews have exclusively focused on depression.

Stage 4: Charting the data

Two researchers not involved in the screening process (CN and WM) will independently review and read the full articles and rate them against the inclusion criteria. Where reviewers do not agree, a consensus meeting will be held to agree on a decision. Throughout all three steps, Cohen's kappa will be calculated to assess the level of agreement between reviewers. If the level of agreement is weak ($\kappa < 0.60$), the process is repeated until there is a strong agreement. This stage aims to contextualise the study findings by briefly describing each article, including the methodology used to reach conclusions [17]. Bibliometric information, including the authors, year of publication, country of study, the study design, study population and essential results, will be captured in Microsoft Excel.

Stage 5: Collating, summarising, and reporting the results

Consistent with a scoping review approach, the extracted data will be collated and presented in a narrative synthesis applying descriptive statistical (tabular supplements) and qualitative methods [22]. The results will be presented thematically in line with the study objectives. We will qualitatively summarise the epidemiology of transdiagnostic depressed mood, its outcome measures, interventions and natural progression in anxiety and psychosis. For instance, through numerical analysis, we will produce charts and tables highlighting the distribution of the findings concerning geographical distribution, distribution across diagnoses, distribution across the years and the nature of the reported studies. The scoping nature of the study requires a qualitative approach. To further illustrate this, we will not perform a meta-analysis to synthesise risk factors associated with depressed mood by calculating a summative effect size; instead, we will qualitatively describe the most reported risk factors.

Stage 6: Consultation exercise

Before compiling a final report, we will present our preliminary findings to various stakeholders, including individuals with lived experience, clinicians, and researchers. The research team will also orient the stakeholders on the scoping review objectives before presenting the preliminary findings. This will help gain more insight into the meaning of the results and guide the research team towards resources and evidence that might have been missed through the search strategy in preparation for the final report. Additionally, this stage will serve as a precursor to disseminating study findings.

For peer review only - http://bmjopen.bmj.com/site/about/guidelines.xhtml

Patient and public involvement

The experiential meaning and impact of depressed mood as a transdiagnostic target can be further explored through the narratives of those with lived experience. We will conduct extensive and iterative processes of consultation and interaction with various stakeholders, including persons with lived experience of anxiety and/or psychosis, to refine the review questions. We will also triangulate insights from our SR with contextual data from key informants, including persons with lived experience, clinicians, and researchers. We will apply purposive sampling to reach information-rich informers and engage in two rounds of focus group discussions and in-depth interviews.

ETHICS AND DISSEMINATION

This study does not require ethical approval as it is a literature review. The results will be submitted for publication in a peer-reviewed journal. All relevant data/materials, including data collection tools, will be submitted as supplementary files when the results are submitted for publication.

For peer review only

Contributors

JMD and EM were primarily responsible for writing the first draft of the protocol. JMD, EM, PN, RBC, TT, BKS, MA, DC, WM and CN were involved in conceptualising the study and editing all protocol manuscript versions. JMD will search the literature and data management. EM, PN, RBC, TDT, BKS, MA, DC, WM and CN will be responsible for article screening, quality assurance, data extraction and qualitative synthesis. MA, WM, CN and DC will provide overall supervision, mentoring and guidance.

Funding

Wellcome Trust (grant number NA).

Competing interests

All the authors declare no competing interests.

Authors' ORCID information

Jermaine M. Dambi (JMD): https://orcid.org/0000-0002-2446-7903
Edwin Mavindidze (EM): https://orcid.org/0000-0001-9849-8932
Primrose Nyamayaro (PN): https://orcid.org/0000-0002-6856-1002
Tariro Dee Tunduwani (TDT): https://orcid.org/0009-0004-8073-7068
Beatrice Kudzaishe Shava (BKS): https://orcid.org/0000-0002-9130-7010
Rhulani Beji-Chauke (RBC): https://orcid.org/0000-0002-3157-5507
Webster Mavhu (WM): https://orcid.org/0000-0003-1881-4398
Melanie Abas (MA): https://orcid.org/0000-0003-1716-5584
Dixon Chibanda (DC): https://orcid.org/0000-0003-2505-8607
Clement Nhunzvi (CN): https://orcid.org/0000-0001-5804-9817

References

1 Wilson RS, Yung AR, Morrison AP. Comorbidity rates of depression and anxiety in first episode psychosis: A systematic review and meta-analysis. *Schizophr Res*. 2020;216:322–9.

2 Saha S, Lim CCW, Cannon DL, *et al.* Co-morbidity between mood and anxiety disorders:

A systematic review and meta-analysis. *Depress Anxiety*. 2021;38:286–306.

3 Lemstra M, Neudorf C, D'Arcy C, *et al.* A systematic review of depressed mood and anxiety by SES in youth aged 10-15 years. *Can J Public Heal*. 2008;99:125–9.

4 Stålner O, Nordin S, Madison G. Six-year prognosis of anxiety and depression caseness and their comorbidity in a prospective population-based adult sample. *BMC Public Health*. 2022;22:1–9.

5 Kaiser T, Herzog P, Voderholzer U, *et al.* Unraveling the comorbidity of depression and anxiety in a large inpatient sample : Network analysis to examine bridge symptoms. *Depress Anxiety*. 2021;38:307–17.

6 Rosellini AJ, Boettcher H, Brown TA, *et al.* A Transdiagnostic Temperament-Phenotype Profile Approach to Emotional Disorder Classification : An Update. 2015;2:110–28.

7 Keynejad R, Spagnolo J, Thornicroft G. WHO mental health gap action programme (mhGAP) intervention guide : updated systematic review on evidence and impact. 2021;124–30.

8 Zarate-Guerrero S, Duran JM, Naismith I. How a transdiagnostic approach can improve the treatment of emotional disorders: Insights from clinical psychology and neuroimaging. *Clin Psychol Psychother*. 2022;29:895–905.

9 Schaeuffele C, Schulz A, Knaevelsrud C, *et al.* CBT at the Crossroads: The Rise of Transdiagnostic Treatments. *Int J Cogn Ther*. 2021;14:86–113.

10 Trust W. Request for Proposals (RFP) for suppliers to review and summarise the current knowledge base for one or more transdiagnostic targets relevant to anxiety , depression and / or psychosis Up to 10 reviews will be commissioned. London 2022. https://cms.wellcome.org/sites/default/files/2022-11/Wellcome-RFP-Transdiagnostics.pdf

11 Liu Q, Joiner RJ, Trichtinger LA, et al. Dissecting the depressed mood criterion in adult depression: The heterogeneity of mood disturbances in major depressive episodes. J Affect Disord. 2023;323:392–9.

12 El-Den S, Chen TF, Gan YL, *et al.* The psychometric properties of depression screening tools in primary healthcare settings: A systematic review. *J Affect Disord*. 2018;225:503–22.

13 Levis B, Benedetti A, Thombs BD. Accuracy of Patient Health Questionnaire-9 (PHQ-9) for screening to detect major depression: Individual participant data meta-analysis. *BMJ*. 2019;365. doi: 10.1136/bmj.l1476

14 Chibanda D, Weiss HA, Verhey R, *et al.* Effect of a primary care-based psychological intervention on symptoms of common mental disorders in Zimbabwe: A randomized clinical trial. *JAMA - J Am Med Assoc*. 2016;316:2618–26.

15 Heissel A, Heinen D, Brokmeier LL, *et al.* Exercise as medicine for depressive symptoms ? A systematic review and meta-analysis with meta-regression. Br J Sports Med 2023;1049–57. doi: 10.1136/bjsports-2022-106282.

16 Noetel M, Sanders T, Gallardo-gómez D, *et al.* Effect of exercise for depression : systematic review and network meta-analysis of randomised controlled trials Summary A systematic review and network meta-analysis. BMJ. 2024;384:e075847. doi: 10.1136/bmj-2023-075847.

17 Arksey H, O'Malley L. Scoping studies: towards a methodological framework. *Int J Soc Res Methodol*. 2005;8:19–32.

18 Peters MDJ, Godfrey C, McInerney P, *et al.* Best practice guidance and reporting items for the development of scoping review protocols. *JBI Evid Synth*. 2022;20:953–68.

19 Tricco AC, Lillie E, Zarin W, et al. PRISMA Extension for Scoping Reviews (PRISMA-ScR): Checklist and Explanation. Ann Intern Med. 2018; 169:467–73.

For peer review only - http://bmjopen.bmj.com/site/about/guidelines.xhtml

20 Samnani SS, Vaska M, Ahmed S, *et al.* Review typology: The basic types of reviews for synthesizing evidence for the purpose of knowledge translation. *J Coll Physicians Surg Pakistan*. 2017;27:635–41.

21 Ouzzani M, Hammady H, Fedorowicz Z, *et al.* Rayyan-a web and mobile app for systematic reviews. *Syst Rev*. 2016;5:1–10.

22 Mallidou A. Mapping the landscape of knowledge synthesis. *Nurs Manage*. 2014;21:30–9.

For peer review only

For peer review only - http://bmjopen.bmj.com/site/about/guidelines.xhtml

Preferred Reporting Items for Systematic reviews and Meta-Analyses extension for Scoping Reviews (PRISMA-ScR) Checklist

SECTION	ITEM	PRISMA-ScR CHECKLIST ITEM	REPORTED ON PAGE #
**TITLE**			
Title	1	Identify the report as a scoping review.	3
**ABSTRACT**			
Structured summary	2	Provide a structured summary that includes (as applicable): background, objectives, eligibility criteria, sources of evidence, charting methods, results, and conclusions that relate to the review questions and objectives.	4
**INTRODUCTION**			
Rationale	3	Describe the rationale for the review in the context of what is already known. Explain why the review questions/objectives lend themselves to a scoping review approach.	3-4
Objectives	4	Provide an explicit statement of the questions and objectives being addressed with reference to their key elements (e.g., population or participants, concepts, and context) or other relevant key elements used to conceptualize the review questions and/or objectives.	5-6
**METHODS**			
Protocol and registration	5	Indicate whether a review protocol exists; state if and where it can be accessed (e.g., a Web address); and if available, provide registration information, including the registration number.	N/A
Eligibility criteria	6	Specify characteristics of the sources of evidence used as eligibility criteria (e.g., years considered, language, and publication status), and provide a rationale.	4-5
Information sources*	7	Describe all information sources in the search (e.g., databases with dates of coverage and contact with authors to identify additional sources), as well as the date the most recent search was executed.	7
Search	8	Present the full electronic search strategy for at least 1 database, including any limits used, such that it could be repeated.	7
Selection of sources of evidence†	9	State the process for selecting sources of evidence (i.e., screening and eligibility) included in the scoping review.	7-8
Data charting process‡	10	Describe the methods of charting data from the included sources of evidence (e.g., calibrated forms or forms that have been tested by the team before their use, and whether data charting was done independently or in duplicate) and any processes for obtaining and confirming data from investigators.	8
Data items	11	List and define all variables for which data were sought and any assumptions and simplifications made.	7-8
Critical appraisal of individual sources of evidence§	12	If done, provide a rationale for conducting a critical appraisal of included sources of evidence; describe the methods used and how this information was used in any data synthesis (if appropriate).	N/A
Synthesis of results	13	Describe the methods of handling and summarizing the data that were charted.	8

For peer review only - http://bmjopen.bmj.com/site/about/guidelines.xhtml

SECTION	ITEM	PRISMA-ScR CHECKLIST ITEM	REPORTED ON PAGE #
**RESULTS**			
Selection of sources of evidence	14	Give numbers of sources of evidence screened, assessed for eligibility, and included in the review, with reasons for exclusions at each stage, ideally using a flow diagram.	n/a
Characteristics of sources of evidence	15	For each source of evidence, present characteristics for which data were charted and provide the citations.	N/A
Critical appraisal within sources of evidence	16	If done, present data on critical appraisal of included sources of evidence (see item 12).	N/A
Results of individual sources of evidence	17	For each included source of evidence, present the relevant data that were charted that relate to the review questions and objectives.	N/A
Synthesis of results	18	Summarize and/or present the charting results as they relate to the review questions and objectives.	N/A
**DISCUSSION**			
Summary of evidence	19	Summarize the main results (including an overview of concepts, themes, and types of evidence available), link to the review questions and objectives, and consider the relevance to key groups.	8-9
Limitations	20	Discuss the limitations of the scoping review process.	N/A
Conclusions	21	Provide a general interpretation of the results with respect to the review questions and objectives, as well as potential implications and/or next steps.	N/A
**FUNDING**			
Funding	22	Describe sources of funding for the included sources of evidence, as well as sources of funding for the scoping review. Describe the role of the funders of the scoping review.	9

JBI = Joanna Briggs Institute; PRISMA-ScR = Preferred Reporting Items for Systematic reviews and Meta-Analyses extension for Scoping Reviews.

* Where *sources of evidence* (see second footnote) are compiled from, such as bibliographic databases, social media platforms, and Web sites.

† A more inclusive/heterogeneous term used to account for the different types of evidence or data sources (e.g., quantitative and/or qualitative research, expert opinion, and policy documents) that may be eligible in a scoping review as opposed to only studies. This is not to be confused with *information sources* (see first footnote).

‡ The frameworks by Arksey and O'Malley (6) and Levac and colleagues (7) and the JBI guidance (4, 5) refer to the process of data extraction in a scoping review as data charting.

§ The process of systematically examining research evidence to assess its validity, results, and relevance before using it to inform a decision. This term is used for items 12 and 19 instead of "risk of bias" (which is more applicable to systematic reviews of interventions) to include and acknowledge the various sources of evidence that may be used in a scoping review (e.g., quantitative and/or qualitative research, expert opinion, and policy document).

From: Tricco AC, Lillie E, Zarin W, O'Brien KK, Colquhoun H, Levac D, et al. PRISMA Extension for Scoping Reviews (PRISMAScR): Checklist and Explanation. Ann Intern Med. 2018;169:467–473. doi: 10.7326/M18-0850.

For peer review only - http://bmjopen.bmj.com/site/about/guidelines.xhtml

S1 Table: Additional search strategies

**Population**	Adults
**Exposure/conditions**	*Depressed mood NOT depression AND (psychosis OR anxiety)
**Interventions**	Any, e.g. pharmacological, psychological, physical, etc<br>* to run a generic approach at first and then run subsequent searches specifying the intervention type
**Comparison**	Any, i.e. control, treatment as usual
**Time**	2004 up to search penultimate date
**Setting**	Unrestricted
**Study designs**	Quantitative, i.e. cross-sectional, case-control, cohort, experimental designs (e.g. clinical trials, quasi-experimental designs)
**Language**	English only

For peer review only
